# Enzymatic Assays for the Diagnosis of Bradykinin-Dependent Angioedema

**DOI:** 10.1371/journal.pone.0070140

**Published:** 2013-08-05

**Authors:** Federica Defendi, Delphine Charignon, Arije Ghannam, Remi Baroso, Françoise Csopaki, Marion Allegret-Cadet, Denise Ponard, Bertrand Favier, Sven Cichon, Brigitte Nicolie, Olivier Fain, Ludovic Martin, Christian Drouet, on behalf of the National Reference Centre for Angioedema CREAK

**Affiliations:** 1 Centre de Référence des Angioedèmes à Kinines, CREAK, Grenoble, France; 2 Université Joseph Fourier, GREPI/AGIM CNRS FRE 3405, Grenoble, France; 3 Universität Bonn, Institut für Humangenetik, Bonn, Germany; 4 Division of Medical Genetics, Department of Biomedicine, University of Basel, Basel, Switzerland; 5 CHU Angers, Département d’Allergologie, Angers, France; 6 Université Paris XIII, AP-HP, Hôpital Jean Verdier, Médecine Interne, Bondy, France; 7 Université L’UNAM, Hôpital d’Angers, Département de Dermatologie, Angers, France; University of Leicester, United Kingdom

## Abstract

**Background:**

The kinins (primarily bradykinin, BK) represent the mediators responsible for local increase of vascular permeability in hereditary angioedema (HAE), HAE I-II associated with alterations of the *SERPING1* gene and HAE with normal C1-Inhibitor function (HAE-nC1INH). Besides C1-Inhibitor function and concentration, no biological assay of kinin metabolism is actually available to help physicians for the diagnosis of angioedema (AE). We describe enzymatic tests on the plasma for diagnosis of BK-dependent AE.

**Methods:**

The plasma amidase assays are performed using the Pro-Phe-Arg-*p*-nitroanilide peptide substrate to evaluate the spontaneous amidase activity and the proenzyme activation. We analyzed data of 872 patients presenting with BK-dependent AE or BK-unrelated diseases, compared to 303 controls. Anti-high MW kininogen (HK) immunoblot was achieved to confirm HK cleavage in exemplary samples. Reproducibility, repeatability, limit of blank, limit of detection, precision, linearity and receiver operating characteristics (ROC) were used to calculate the diagnostic performance of the assays.

**Results:**

Spontaneous amidase activity was significantly increased in all BK-dependent AE, associated with the acute phase of disease in HAE-nC1INH, but preserved in BK-unrelated disorders. The increase of the amidase activity was associated to HK proteolysis, indicating its relevance to identify kininogenase activity. The oestrogens, known for precipitating AE episodes, were found as triggers of enzymatic activity. Calculations from ROC curves gave the optimum diagnostic cut-off for women (9.3 nmol⋅min^−1^⋅mL^−1^, area under curve [AUC] 92.1%, sensitivity 80.0%, and specificity 90.1%) and for men (6.6 nmol·min^−1^⋅mL^−1^, AUC 91.0%, sensitivity 87.0% and specificity 81.2%).

**Conclusion:**

The amidase assay represents a diagnostic tool to help physicians in the decision to distinguish between BK-related and –unrelated AE.

## Introduction

Kinin-mediated angioedema (AE) is an uncommon disorder characterized by recurrent and unpredictable episodes of localized swelling (face, extremities, bowel wall, genitals and upper airways), due to increased vascular permeability in subcutaneous and submucosal tissues [1], [2]. Occurring at the endothelium/plasma interface, this phenomenon depends on kinins (bradykinin [BK], *des*Arg^9^-BK), released upon proteolytic activities on the high MW kininogen (HK) during the contact-system activation [1], [2], [3], [4]. Plasma kallikrein mainly supports this proteolytic activity [5].

Kinin-mediated AE can be inherited (HAE), acquired (AAE) or iatrogenic (*e.g*. induced by angiotensin-converting enzyme inhibitors). Inadequate control of the contact phase activation and subsequent kinin overproduction give rise to HAE [2], [4], [6] primarily attributed to a genetic deficiency (HAE I) or dysfunction (HAE II) of C1-Inhibitor (C1INH). AAE is mainly associated with B-cell lymphoproliferative diseases, or autoimmune and neoplastic diseases [7], [8]. HAE I–II and AAE diagnoses are based on the clinical picture in combination with a laboratory diagnosis demonstrating reduced C1INH function (<50%) associated or not with low levels of C1INH and C4 [9]. We [10] and others [11], [12] have described HAE with normal C1INH function (HAE-nC1INH) manifesting as recurrent AE with possible association with *F12* gene mutations [13], [14]. We previously demonstrated that these plasmas transiently develop an important increase of amidase activity which was initially qualified as a gain-of-function of factor XII [13], [15]. However, the absence of mutation in *F12* gene in many patients rises the hypothesis that HAE-nC1INH actually refers to several conditions [16]. HAE-nC1INH is currently diagnosed on the observation of clinical manifestations reminiscent of HAE and positive family history. The disease is often underdiagnosed, or overdiagnosed, because of its non-specific phenotype as well as variable severity and penetrance. When the *F12* gene is normal, the absence of biological diagnosis is a true limit for the clinical practice and management of a majority of patients suffering from presumable HAE-nC1INH.

Evidence for contact system involvement in HAE-nC1INH comes from *ex vivo* studies that demonstrated its activation in plasma of patients [13], [16], and evidence for BK formation comes from successful treatment with a B2 receptor antagonist [17]. However, circulating kinins are short-lived peptides (27±10 sec) and circulate at low concentrations (15–90 pM during attacks). This prompted us to investigate the BK production enzymes as stable and accessible parameters, rather than labile blood peptides.

The burden of HAE-nC1INH and the efforts to properly diagnose and treat the disease are substantial [16]. The present paper develops the amidase assays relevant to accurate and meaningful biological diagnosis of BK-AE. In addition to immediate diagnostic outcomes of interest for physicians, the data argue that the BK-AE illustrated herein refer to conditions of contact phase activation.

## Patients and Methods

### 1. Patients

#### 1.1 Ethics statement

All procedures were performed according to the principles expressed in the Helsinki declaration and to the French ethical policies for the proper execution of this study: anonymous biological sample collection (Ministry of Health identification DC-2008-634), written consent to participate in the genetic investigation with associated biological assays. Blood donors (healthy controls) answered a questionnaire; when the genetic investigation was not developed (IgE-AE, HD-AE and inflammatory patients), the individuals provided oral informed consent to their physicians (LM, BN, OF) in giving the permission to use samples and in being informed of the final results of the study. The Institutional Review Board of Grenoble (South-East committee V) stated that sample collection and its processing agreed with these ethics requirements (decision April 1st 2009). In addition, this is in compliance with the “Informatique & liberté” act under the ID 909453. All the data were analyzed anonymously.

#### 1.2 Patients and sampling procedures

The details are presented in Supporting Information section S1 in File S1.

#### 1.3 Plasma amidase assays

Plasma samples were prepared from citrate-blood collections, centrifuged to prepare the platelet-free plasma and immediately frozen at −80°C. The spontaneous amidase activity was evaluated using the peptide substrate HD-Pro-Phe-Arg-*p*NA (1 mM; Bachem), representing the P1-P′1 scissile bond by kallikrein at the C-terminus of BK. This assay refers to enzymes with spontaneous amidase activity, *i.e*. the Serine proteases of contact phase and fibrinolysis (kallikrein, FXII, plasmin and tissue-type plasminogen activator). Spontaneous amidase activity was kinetically monitored by the A_405_ at 30°C (ThermoFisher Spectrophotometer), and expressed in nmol⋅min^−1^⋅mL^−1^ (molar extinction coefficient of *p*-nitroaniline 8800 M^−1^⋅cm^−1^).

In order to refer to plasma proenzyme activation, the contact system was activated by cold pre-incubation of plasma sample with dextran sulfate (12.5 mg⋅mL^−1^) [18], then the subsequent enzyme activity was assessed using the peptide substrate HD-Pro-Phe-Arg-*p*NA and monitored by the A_405_ at 30°C.

#### 1.4 Gel electrophoresis and immunoblot analysis

The details are presented in Supporting Information section S2 in File S1.

### 2. Performances of the Amidase Assays

#### 2.1 Reproducibility, repeatability, limit of blank, limit of detection, precision and linearity

Reproducibility of the amidase assays was evaluated by testing 3 distinct lots of the internal plasma sample control, on different days and by different analysts. The median and the coefficient of variation (CV) were computed for spontaneous amidase activity and proenzyme activation. The repeatability was assessed by testing 5 samples (3 reference positive and 2 reference negative samples) 5 times, on the same day and by the same analyst. The median and CV were computed for spontaneous amidase activity and proenzyme activation. The limit of blank and the limit of detection were calculated as described [19]. The precision was assessed by testing 4 samples (2 reference positive and 2 reference negative samples) 5 times in 5 different assays. The CV is calculated for each sample, for spontaneous amidase activity and proenzyme activation. The linearity under dilution was assessed using a preparation of standard kallikrein (Enzyme Research, Swansea, UK).

#### 2.2 Receiving Operator Characteristics (ROC)

The diagnostic performance of the amidase assay for AE disease was evaluated by analysis of Receiver Operating Characteristics (ROC) curves (XLSTAT software; Addinsoft™). Then were calculated cut-off, sensitivity, specificity, positive and negative predictive value (PPV and NPV) and the corresponding area under the curve (AUC) with 95% confidence intervals (CI). Considering the hormone influence on the disease [12], [20], [21], two distinct data were presented for women and men.

### 3. Statistical Analyses

All data were represented as means ±SEM. Kruskal-Wallis with Dunn’s multiple comparison, Mann-Whitney or Student’s *t* tests were performed to assess statistical significance. *P*-values <0.05 were considered statistically significant.

## Results

### 1. Patients

We collected citrate plasma samples from patients diagnosed with HAE I-II (n = 250; 217 HAE I, 33 HAE II; 95 males/155 females), with AAE (n = 20; 8 males/12 females), and with HAE-nC1INH during the attacks and the remission periods (60 *F12* mutation carriers, 7 males/53 females; 268 non-carriers, 82 males/186 females). A large group of the women (64/239, 27%) suffering from HAE-nC1INH reported worsening of symptoms during oestrogen contraceptive (OC) therapy (n = 57) and/or pregnancy (n = 7). We also collected control samples from individuals presenting with documented IgE-dependent allergic AE (IgE-AE, n = 64), non-allergic idiopathic histamine-dependent AE (HD-AE, n = 62) and various chronic inflammatory disorders (n = 23).

### 2. Performances of the Amidase Assay

#### 2.1 Reproducibility, repeatability, limit of detection, precision and linearity

By testing the reproducibility the median values obtained for spontaneous amidase activity were 9.0 nmol⋅min^−1^⋅mL^−1^ (CV = 27.9%), 7.2 nmol⋅min^−1^⋅mL^−1^ (CV = 26.7%) and 9.3 nmol⋅min^−1^⋅mL^−1^ (CV = 27.9%). The median values obtained for proenzyme activation assay were 2047 nmol⋅min^−1^⋅mL^−1^ (CV = 7.8%), 2126 nmol⋅min^−1^⋅mL^−1^ (CV = 8.3%) and 2084 nmol⋅min^−1^⋅mL^−1^ (CV = 4.5%).

We next test the repeatability; the median values obtained for spontaneous amidase assay were 13.9 nmol⋅min^−1^⋅mL^−1^ (CV = 16.5%), 10.4 nmol⋅min^−1^⋅mL^−1^ (CV = 2.6%), 9.8 nmol⋅min^−1^⋅mL^−1^ (CV = 15.9%), 6.3 nmol⋅min^−1^⋅mL^−1^ (CV = 3.5%), and 6.2 nmol⋅min^−1^⋅mL^−1^ (CV = 4.7%). The median values obtained for proenzyme activation assay were 2147 nmol⋅min^−1^⋅mL^−1^ (CV = 5.0%), 2027 nmol⋅min^−1^⋅mL^−1^ (CV = 7.5%), 2119 nmol⋅min^−1^⋅mL^−1^ (CV = 4.5%), 2050 nmol⋅min^−1^⋅mL^−1^ (CV = 1.8%), 2156 nmol⋅min^−1^⋅mL^−1^ (CV = 2.2%). The limit of blank was 0.25 nmol⋅min^−1^⋅mL^−1^ and the limit of detection was 0.99 nmol⋅min^−1^⋅mL^−1^ for both spontaneous amidase and proenzymes activation assays. The precision (CV) values were 23.56%, 24.42%, 28.43%, and 23.80% for spontaneous amidase activity and 5.13%, 3.12%, 3.00% and 3.47% for proenzymes activation. The linearity under dilution of a standard kallikein preparation resulted in linear regression equations with correlation coefficient of 0.9960.

#### 2.2. Sensitivity, Specificity, Predictive Values and ROC curve

**Figure 1:**
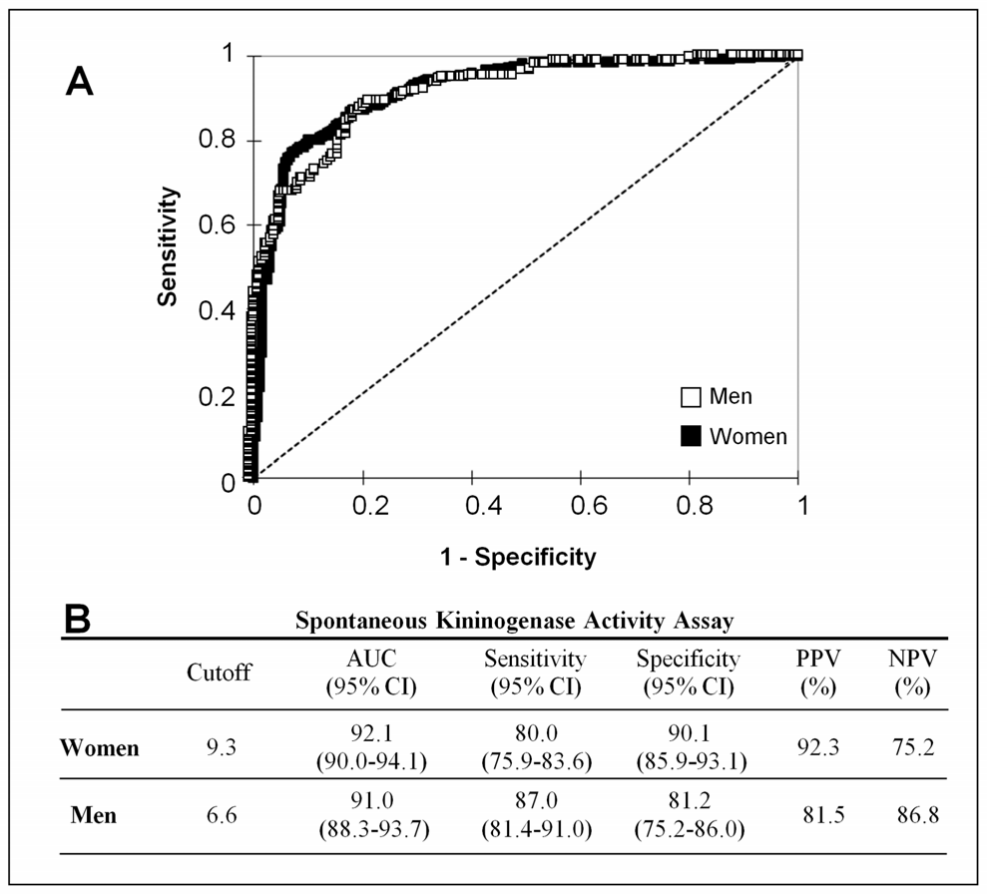
Diagnostic outcomes for spontaneous amidase assay in the diagnosis of BK-dependent AE. (A) Receiving operator characteristic (ROC) curves of male and female individuals. (B) Diagnostic values for spontaneous amidase activity. Data were generated from women (n = 678: HAE I-II, n = 155; HAE-nC1INH carriers of the *F12* mutation, n = 53; HAE-nC1INH non-carriers, n = 186; AAE, n = 12; asymptomatic HAE-I, n = 5; asymptomatic HAE-nC1INH non-carriers of the *F12* mutation, n = 63; IgE-mediated AE, n = 50; HD-AE, n = 39; chronic inflammatory disorders, n = 14; healthy donors, n = 101) and from men (n = 394: HAE I-II, n = 95; HAE-nC1INH carriers of the *F12* T928K mutation, n = 7; HAE-nC1INH non-carriers, n = 82; AAE, n = 8; asymptomatic HAE-I, n = 2; asymptomatic HAE-nC1INH non-carriers of the *F12* mutation, n = 55; IgE-mediated AE, n = 14; HD-AE, n = 23; chronic inflammatory disorders, n = 9; healthy donors, n = 99).

We evaluated the performance of the amidase assay using ROC analysis. ROC curves showed the optimum diagnostic cut-off for spontaneous amidase assay for women was 9.3 nmol⋅min^−1^⋅mL^−1^ (AUC 92.1%, sensitivity 80.0%, specificity 90.1%). The optimum cut-off value for men was 6.6 nmol⋅min^−1^⋅mL^−1^ (AUC 91.0%, sensitivity 87.0%, specificity 81.2%) (Fig. 1A and 1B). Predictive values for the spontaneous amidase assay in the diagnosis of BK-AE are shown in Fig. 1B. Both indicators of performance for diagnosis of AE disease are consistent with high excellence in the scale for AUC and good for sensitivity and specificity.

### 3. Amidase Activity for AE Diagnosis

**Figure 2:**
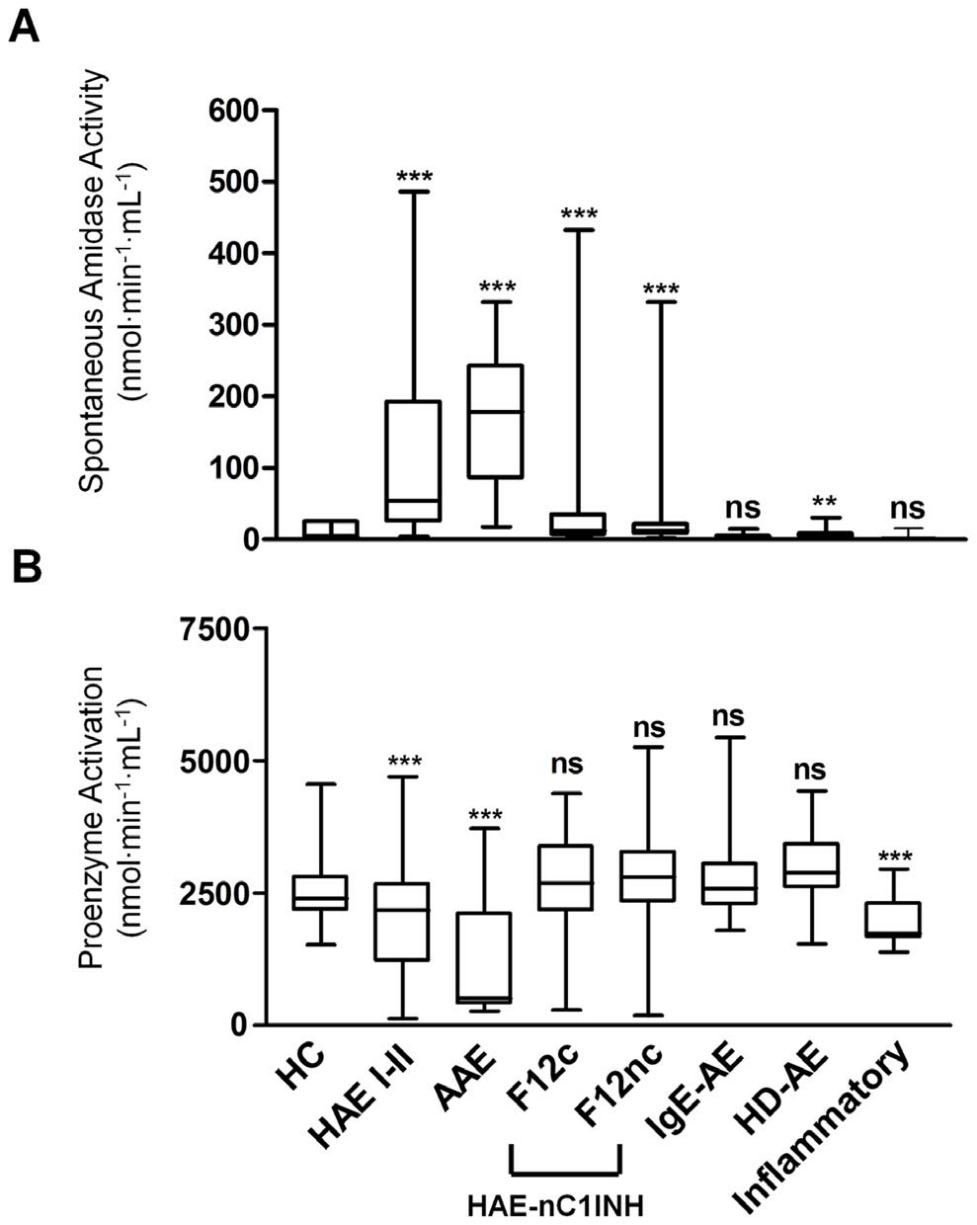
Distribution of amidase activities in AE situations. (A) Plasma spontaneous amidase activity and (B) proenzyme activation in healthy controls (HC, n = 303), patients with HAE I-II (n = 250), AAE (n = 20), HAE-nC1INH carriers of *F12* mutations (F12c, n = 60) or non-carriers (F12nc, n = 268), patients suffering from IgE-mediated angioedema (IgE-AE, n = 64), non-allergic idiopathic histamine-dependent angioedema (HD-AE, n = 62) or chronic inflammatory diseases (Inflammatory, n = 23). Graph labels are identical for panel A and B. ns, not significant; ***, *P*<0.001; **, *P*<0.01 *vs* healthy control group. Box-whisker plots show the median (horizontal bars within boxes), the interquartile range (boxes) and the 5^th^ to 95^th^ centiles (vertical bars).

Amidase activities were assayed in HAE I-II, AAE, and HAE-nC1INH (*F12* mutation carriers, and non-carriers). Spontaneous amidase activity was found significantly increased in plasma of patients identified as HAE I-II, AAE, and HAE-nC1INH with or without *F12* mutation (110.6±7.2, 168.1±21.9, 63.1±13.9 and 32.4±3.7 nmol⋅min^−1^⋅mL^−1^, respectively; *P*<0.001 for each AE group *vs* controls) compared with amidase activity in healthy controls (4.2±0.2 nmol⋅min^−1^⋅mL^−1^; n = 303) (Fig. 2A). The spontaneous amidase activity was found elevated in HAE I-II and AAE whatever the period, symptomatic or not.

The proenzyme activation was found strongly decreased in HAE I-II and AAE (1967.0±58.6 and 1216.0±269.4 nmol⋅min^−1^⋅mL^−1^, respectively) compared to healthy controls (2548.0±32.1 nmol⋅min^−1^⋅mL^−1^) (*P*<0.001 for both AE situations) (Fig. 2B). In HAE-nC1INH patients, proenzyme levels were found preserved (2559.0±143.3 and 2814.0±56.6 nmol⋅min^−1^⋅mL^−1^ for *F12* mutation carriers and non-carriers, respectively) compared to control group (2548.0±32.1 nmol⋅min^−1^⋅mL^−1^) (Fig. 2B).

We also tested plasma samples from individuals with documented IgE-AE, HD-AE and inflammatory chronic disorders. In IgE-AE, the spontaneous amidase activity was comparable to control (4.5±0.3 and 4.2±0.2 nmol⋅min^−1^⋅mL^−1^, respectively) (*P*>0.05) (Fig. 2A). The proenzyme activation was not decreased compared to control (2738.0±87.8 and 2548.0±32.1 nmol⋅min^−1^⋅mL^−1^, respectively) (Fig. 2B). Taken together, these results indicate that, as expected, contact phase-dependent kinin formation was not implicated in IgE-AE. In patients suffering from HD-AE, the spontaneous amidase activity was increased (6.9±0.7 nmol⋅min^−1^⋅mL^−1^) compared to healthy control (4.2±0.2 nmol⋅min^−1^⋅mL^−1^; *P*<0.01) (Fig. 2A). This suggests that kinin formation enzymes may develop during mast cell activation with the hypothesis of a partial involvement of BK in the pathogenesis of this disorder. Proenzyme activation capacity was found preserved (2951.0±79.4 nmol⋅min^−1^⋅mL^−1^) compared to control (2548.0±32.1 nmol⋅min^−1^⋅mL^−1^) (Fig. 2B). In inflammatory situations (Inflammatory; chronic infection, autoimmunity, chronic inflammatory disorders susceptible to corticosteroids and NSAIDs ), the spontaneous amidase activity was similar to control (3.6±0.6 and 4.2±0.2 nmol⋅min^−1^⋅mL^−1^, respectively) (*P*>0.05) (Fig. 2A); however proenzyme activation was significantly decreased (1885.0±88.1 nmol⋅min^−1^⋅mL^−1^) compared to control (2548.0±32.1 nmol⋅min^−1^⋅mL^−1^) (*P*<0.001) suggesting that the zymogens are activated by endothelial stress induced upon the inflammatory disorders.

### 4. Amidase Activity and the AE Disease Expression

**Figure 3:**
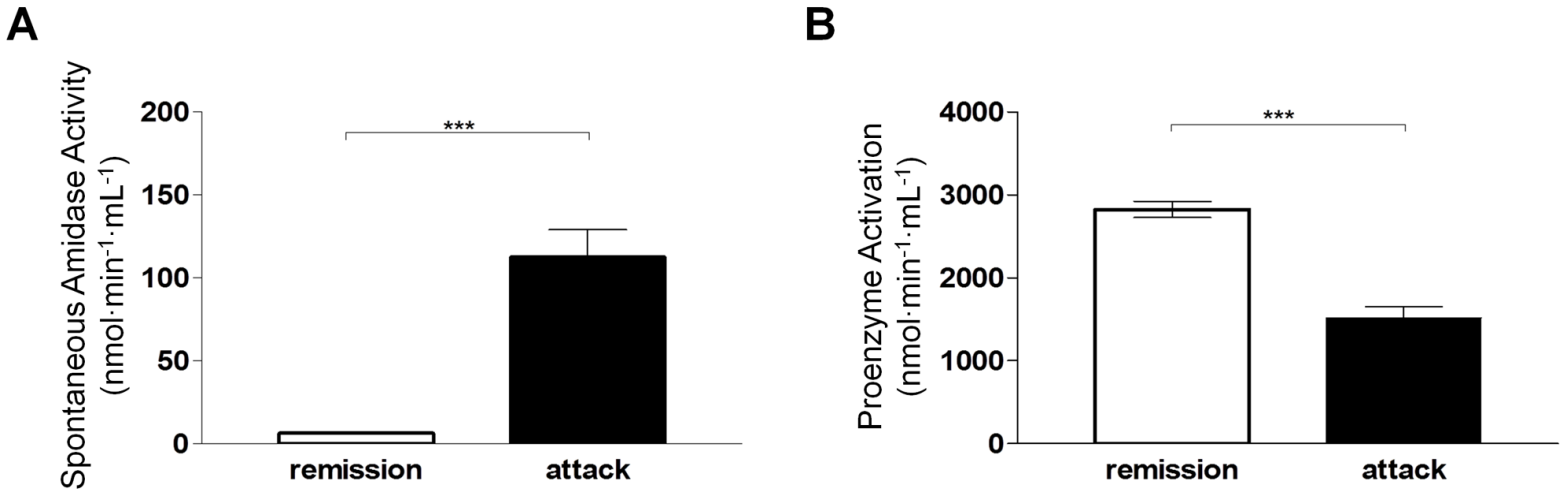
Amidase activities and disease expression. (A) Plasma spontaneous amidase activities and (B) proenzyme activation in patients suffering from HAE-nC1INH (6 carriers and 38 non-carriers of *F12* mutation, n = 44) during remission or attack periods. Remission period refers to >48 hrs after symptoms disappearance; attack period refers to <24 hrs after symptom onset. ***, *P*<0.0001.

Kinin formation is attributed to uncontrolled activation of contact phase proenzymes depending on the endothelial stress conditions (infection/inflammation, age, physical trauma, etc) drug administration (*e.g*. oestrogen). Consequently, the measurements of spontaneous amidase activity and proenzyme activation could be influenced by disease expression. In order to evaluate the performance of the assay in the course of the pathological scenery, we compared spontaneous amidase activity and proenzyme activation from patients suffering from HAE-nC1INH (6 carriers and 38 non-carriers of *F12* mutation), during both remission and attack phases (>48 hrs after symptoms relief and <24 hrs after onset, respectively). As shown in Fig. 3A, when collected during the acute episodes, the samples display a significant increase of spontaneous amidase activity (112.5±16.6 nmol⋅min^−1^⋅mL^−1^) compared to those during the remission period (6.3±0.6 nmol⋅min^−1^⋅mL^−1^) (*P*<0.0001). During attacks, this high plasma spontaneous enzymatic activity was associated with a significant decrease of the proenzyme activation (Fig. 3B, 1520.0±133.1 nmol⋅min^−1^⋅mL^−1^) comparatively to that was observed during the remission phase (2825.0±95.4 nmol⋅min^−1^⋅mL^−1^) (*P*<0.0001).

### 5. Impact of Tranexamic Acid on the Amidase Activity

Tranexamic acid (TA), an antifibrinolytic drug, prevents the plasmin-dependent amplification of BK formation by inhibiting the conversion of plasminogen to plasmin; this drug is currently used for prophylaxis or acute treatment of BK-dependent AE [22].

**Figure 4:**
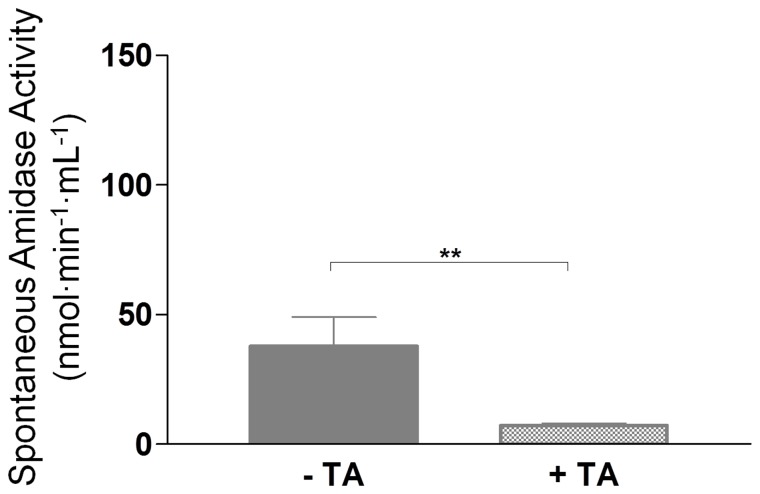
Tranexamic acid (TA) and amidase activity. Plasma spontaneous amidase activity of HAE-nC1INH patients before (– TA) and during (+ TA) treatment (n = 38). **, *P*<0.01.

In order to investigate the impact of TA on kinin forming activity, plasma from a group of 38 patients suffering from HAE-nC1INH and not carrying *F12* mutation was investigated for spontaneous amidase activity before and during TA prophylaxis (1–4 g/day) (Fig. 4). The prophylactic treatment correlated with improvement of clinical symptoms or remission and significant decrease of amidase activity (37.8±11.3 to 7.1±0.9 nmol⋅min^−1^⋅mL^−1^; *P*<0.05).

### 6. Oestrogens as Triggers of Amidase Activity

**Figure 5:**
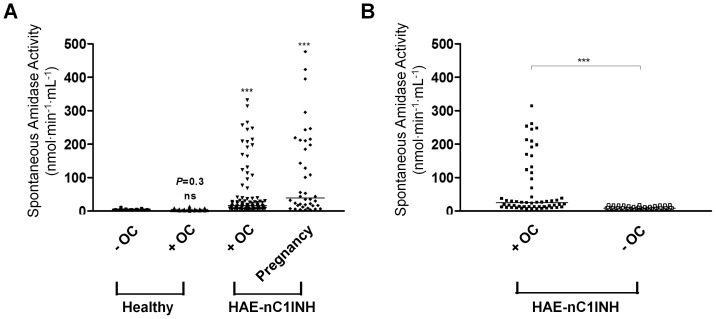
Oestrogen intake and spontaneous amidase activity. (A) Plasma spontaneous amidase activities in female blood donors not taking oestrogen combined contraceptive pill (Healthy – OC, n = 37), taking OC (Healthy+OC, n = 49), HAE-nC1INH women with OC (HAE-nC1INH+OC, n = 93) or during pregnancy (HAE-nC1INH Pregnancy, n = 40). ns, not significant; ***, *P*<0.0001 *vs* Healthy – OC. (B) Plasma spontaneous amidase activities in HAE-nC1INH women during OC contraception (+ OC) and after stopping OC pill (– OC); n = 55. ***, *P*<0.0001 *vs* Healthy – OC. The horizontal bars show the median values.

As already described, HAE I-II and HAE-nC1INH women suffer from episodes of AE precipitated or worsened by high oestrogen levels (*e.g.* oestrogen contraceptive [OC] or pregnancy) [14], [15]. This prompted us to investigate the impact of oestrogen on the spontaneous amidase activity in both normal and pathological situations. In female controls taking OC (Healthy+OC) and without OC (Healthy – OC), the enzymatic activity values were comparable (3.4±0.3 and 2.9±0.3 nmol⋅min^−1^⋅mL^−1^; respectively) and no significant difference was found (*P = *0.3) (Fig. 5A). Conversely, in HAE-nC1INH women taking OC and during pregnancy, the plasma spontaneous enzymatic activity was significantly increased (52.3±8.2 and 108.0±20.2 nmol⋅min^−1^⋅mL^−1^, respectively), compared to control group (Healthy+OC, 3.4±0.3 nmol⋅min^−1^⋅mL^−1^; *P*<0.0001 for both) (Fig. 5A). This indicates that the disease precipitated by oestrogen is associated with the increased spontaneous amidase activity.

We next evaluated the relationship between increased amidase activity and triggering oestrogen exposure by measuring spontaneous amidase activity in HAE-nC1INH women before and after withdrawal of OC (>3 months; n = 55). Spontaneous amidase activity was high during OC (66.6±11.4) and significantly decreased after withdrawal of the pill (9.0±0.6 nmol⋅min^−1^⋅mL^−1^; *P*<0.0001) (Fig. 5B). This demonstrates that the oestrogen trigger took part in the increased plasma amidase activity.

### 7. Association of Increased Amidase Activity and Kininogen Cleavage

During acute phases of AE, the plasma kinin-forming activation leads to HK cleavage by active proteases [16], [21], [23], [24], [25].

We further asked whether the increased amidase activity developed in AE would be associated with the proteolytic cleavage of HK and subsequent BK formation. HK proteolysis was investigated on plasma samples from representative patients with HAE I, HAE-nC1INH not carrying *F12* mutation, during OC (HAE-nC1INH+OC) and after withdrawal of the pill (HAE-nC1INH – OC) and IgE-AE, as representative examples of the cohort above described.

**Figure 6:**
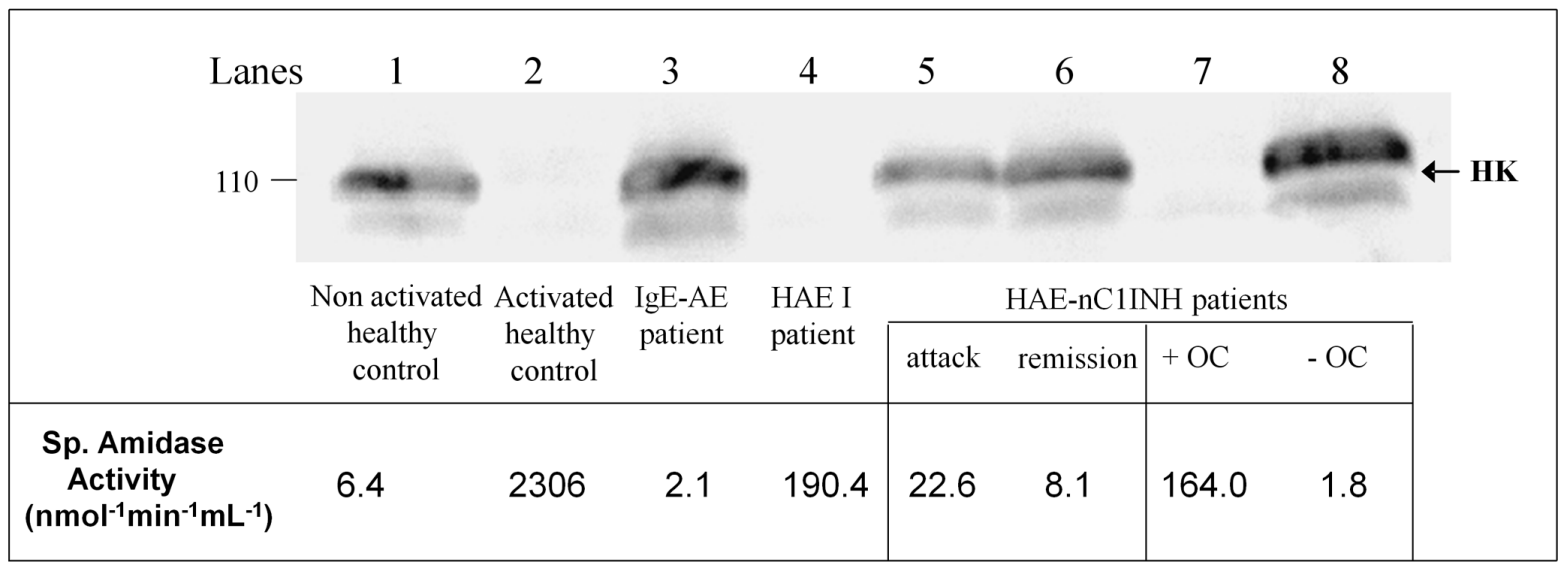
HK cleavage demonstrated by anti-HK L chain immunoblot. Plasma samples were collected from a healthy control, an IgE-dependent angioedema individual (IgE-AE) and patients presenting with HAE I, HAE-nC1INH non-carrier of *F12* mutation (during an attack and the remission period), and with HAE-nC1INH non-carrier of *F12* mutation during OC (+ OC) and after stopping OC pill (– OC) intakes. The values of the corresponding spontaneous amidase activities are indicated.

As shown in Fig. 6, in non-activated donor plasma (lane 1), HK was found under the native form and in agreement with normal enzymatic activity; dextran-sulfate activation of plasma from this control triggered complete cleavage of HK (lane 2) consistently with contact phase activation (positive control). The plasma from an IgE-mediated AE individual displayed the control phenotype (lane 3). HK was also partially processed in plasma from HAE I and from HAE-nC1INH patients during acute disease and fully cleaved in plasma from HAE-nC1INH patient with OC intake (lanes 4, 5 and 7). As illustrated by Fig. 6 in exemplary AE situations, the high spontaneous amidase activity is associated with the HK cleavage, supporting amidase activity as kininogenase activity.

## Discussion

Bradykinin overproduction occurs in pathological conditions where kinin formation is uncontrolled (HAE I-II) or when the enzymatic activity of factor XII or of another hypothetical protease is increased (HAE-nC1INH). The present study intends the testing of patients presenting with BK-AE. This aims to support the distinction between BK-related and –unrelated situations. The increased spontaneous amidase activity was found strongly associated with the active phase for HAE-nC1INH patients and independent on the periods of attack for HAE I-II patients. Besides, in HAE I-II and AAE, two prototypical situations of angioedema, the proenzyme activation was found strongly decreased. These observations are consistent with a contact phase activation with zymogen consumption in the context of the failed control capacity of C1INH [23], a situation which can be distinguish from the HAE-nC1INH condition where the proenzyme activation values were found normal. The spontaneous amidase activity is actually increased during attacks (Fig. 3A), but it could be unexpectedly low due to inadequate timing of plasma collection (*i.e.* after the symptom relief). An abundant HK cleavage was associated with the increased enzymatic activity, supporting the kininogenase activity. This observation agrees with BK overproduction during attacks and puts forward the interest of this assay for BK-AE diagnosis. High values of amidase activity have been reported in individuals presenting with AE [13], [15], [19]. The present evaluation of the assay confirms its suitability to AE diagnosis with good performance indicators, *e.g.* sensitivity, specificity, positive and negative predictive values. In respecting the requirements of the short pre-analytical time lag and optimal sample collection during the active period of the disease, this assay can be recommended in clinical practice.

Patients with C1INH deficiency presented high amidase activity at any time, whatever the level of C1INH function. Most often the samples collected from F12Lys^328^ carriers exhibit pathological values. Patients with HD-AE develop increased amidase activity. This observation suggests the contribution of mast-cell mediators (*i.e*. tryptase, heparine) in triggering BK-mediated AE [26], [27]. Moreover, the mast-cells express B2 receptors [28] with mast-cell degranulation by BK [29] providing a combination of histamine- and BK-mediated mechanisms for this situation. Otherwise, as shown by Fig. 2A, all the unequivocally established IgE-dependent situations display enzymatic activities comparable to controls. This specificity is of high interest in providing the physician with the assistance to distinguish between IgE-mediated- and BK-AE.

HAE patients are known to be sensitive to endogenous or exogenous oestrogens irrespective of the HAE type, with the onset or exacerbation of attacks often linked to exogenous oestrogens or pregnancy [17], [20], [29], [30]. Oestrogen has been attributed to modulate *F12* gene transcription through promoter enhancer sequences [31]. This agrees with the enhanced plasma amidase activity observed after OC intake or during pregnancy in cases of HAE-nC1INH patients, but not in healthy controls (Fig. 5A and -B). This emphasizes the uncommon presentation of HAE-nC1INH, with the high disease frequency in women and precipitation of attacks by oestrogen contributing to the clinical diagnosis [16].

Importantly and in line with the expectations of patients and their physicians, this study makes feasible the biological diagnostic of HAE-nC1INH to the medical community interested in AE, congruent with prior reports of the disease [13], [15]. Combined to *F12* genotyping, the amidase assay will be helpful as a screening diagnostic test for patients with ambiguous recurrent AE and for treatment options, *i.e*. the TA administration.

## Supporting Information

File S1 (DOCX)
