# Correction: Enzymatic Assays for the Diagnosis of Bradykinin-Dependent Angioedema

**DOI:** 10.1371/journal.pone.0100345

**Published:** 2014-06-10

**Authors:** The PLOS ONE Staff

Laurence Bouillet, Isabelle Boccon-Gibod, and Anne Pagnier are mentioned under the Acknowledgements section of the article. Drs Bouillet, Boccon-Gibod, and Pagnier did not consent to being named under this section and as a result their names should be removed and the Acknowledgements section should read as follows:

We are grateful to people of CREAK, the French National Centre for Angioedema, and other people for useful discussions and participation to the collaborative study : M Bergmann, Geneva; T Caballero, Madrid; A Bygum, Odense; K Djenouhat, Algiers; O Michel, Brussels; M Vigan and F Pelletier, Besançon; S Guez, Bordeaux; Y Ollivier, Caen; D-A Moneret-Vautrin and E Beaudouin, Epinal; M Bouvier and B Coppéré, Lyon; H Maillard, Le Mans; D Launay and I Pruvost, Lille; J-J Grob and S Gayet, Marseille; N Raison-Peyron, Montpellier; G Kanny, Nancy; A Gompel, Paris; F Tron, Rouen; S Geny, Strasbourg; D Vincent, Nimes. The authors are indebted to Pr Joel Lunardi and Dr Nicole Monnier, French Reference Centre for Angioedema CREAK Grenoble, for molecular genetics ofSERPING1 and F12 genes, to Hélène Humeau, Angers, for her skillful work and to Dr MV Chuong Nguyen for critical reading of the manuscript.
