# Supplementary material for: Enzymatic Assays for the Diagnosis of Bradykinin-Dependent Angioedema
Source: PLoS One. 2013 Aug 5;8(8):e70140. doi: 10.1371/journal.pone.0070140 (PMC3734293; doi:10.1371/journal.pone.0070140)
Supplement: File S1 — (DOCX) [file pone.0070140.s001.docx]

**SUPPORTING INFORMATIONS**

**S1. Patients and sampling procedures:**

S1.1 Patients

The hereditary angioedema type I-II (HAE I-II) patients were diagnosed on a history of recurrent AE, low concentration and function (<50%) of C1INH protein (HAE I), normal or high concentrations of a non-functional C1INH protein (HAE II). HAE diagnosis was confirmed by demonstrating the mutation in the *SERPING1* gene for all patients.

The diagnosis of acquired angioedema (AAE) was based on low C1INH concentrations and/or diminished C1INH function**,** no family history and in some instances the presence of high level of anti-C1INH antibodies.

The diagnosis of hereditary angioedema with normal C1INH function (HAE-nC1INH) was established on the combination of the following criteria:

- clinical features suggestive of BK-AE (recurrent episodes of swelling lasting at least 24 hrs at various sites including laryngeal) validated by trained physicians
- ineffectiveness of antihistamines and steroids during attacks
- ineffectiveness of antihistamines and/or effectiveness of TA for long term prophylaxis
- absence of profound C1INH deficiency (C1INH function >50%)
- family history

All HAE-nC1INH patients underwent genetic investigation for the missense mutation within the exon 9 of the *F12* gene (10, 14). HAE-nC1INH patients have been checked for normal kinin catabolism by measurement of Aminopeptidase P, Angiotensin-I Converting Enzyme and Carboxypeptidase N activities as previously described (31). All individuals identified with kinin catabolism deficiency (n=229) were excluded from the present study.

The diagnosis of IgE-mediated AE was based on the identification of the responsible allergen by blood testing for specific IgE, skin testing for immediate hypersensitivity (specific prick tests) and/or cautious oral re-challenge.

Non allergic idiopathic histamine-dependent AE (HD-AE) was diagnosed in patients with undisputable chronic or recurrent AE with or without urticaria (spontaneous chronic urticaria) and no allergen identified after careful anamnesis and blood and skin testing. Positive results of skin or blood testing in atopic patients were ignored if these tests had no clinical relevance for AE.

Inflammatory disorders referred to patients without AE but suffering from chronic infection (*Toxocara canis, Helicobacter pylori, HIV*) or chronic inflammatory disorders susceptible to corticosteroids and NSAIDs.

S1.2 Healthy controls

Three-hundred and three healthy individuals (144 men, 159 women) served as normal controls, including female blood donors known for taking oestrogen contraceptive (OC) pill (n=45) or not (n=54).

S1.3 Sampling procedures

Pre-analytical assays have shown that the proenzyme/enzyme system is preserved during 48 hrs at room temperature (20-25°C). The citrate blood samples must be shipped to the laboratory at 20-25°C within 48 hrs. Citrate plasma samples were prepared from patients and healthy controls by centrifugation of freshly collected blood at 2000 × g for 10 min to harvest the platelet-free plasma. The samples were immediately aliquoted and stored at -80°C until further investigation.

**S2. Gel electrophoresis and immunoblot analysis**

Plasma proteins were separated on 8% SDS polyacrylamide gels and then transferred to nitrocellulose membranes at 30 mA during 1h. The electroblot was incubated overnight in blocking buffer [1% BSA, 0.1% Tween 20, 0.15 M NaCl, and 10 mM Tris-HCl, pH 7.5]. Then the membrane was incubated with HRP-conjugated anti-goat anti-HK L chain antibody (Enzyme Research, Swansea, UK; 1:10,000 dilution) at room temperature during 1h. HK was detected by enhanced chemiluminescence (Amersham, Les Ulis, France).
